# Mogrosides as Dual-Function Sweeteners: A Comprehensive Review of Extraction, Metabolism, Antidiabetic Mechanisms, and Food Applications

**DOI:** 10.3390/nu18091342

**Published:** 2026-04-24

**Authors:** Qiqi Feng, Tao Wang, Qing Wang, Hongyu Pan, Yujie Zhong, Yongliang Zhuang

**Affiliations:** 1 Faculty of Food Science and Engineering, Kunming University of Science and Technology, Kunming 650500, China; fengqiqi2024@163.com (Q.F.);; 2 Yunnan Technology Innovation Center of Woody Oil, Kunming 650201, China; 3 Yunnan Key Laboratory of Plateau Food Advanced Manufacturing, Kunming 650500, China

**Keywords:** mogrosides, extraction, metabolism, antidiabetic activity, food industry

## Abstract

Mogrosides, the primary bioactive compounds of *Siraitia grosvenorii*, are natural, non-caloric sweeteners with promising therapeutic potential for diabetes. They provide a dual advantage: delivering sweetness without impacting blood glucose levels, while simultaneously exerting beneficial antidiabetic effects. This review systematically synthesizes current knowledge on mogrosides, covering their extraction methods, metabolic pathways, and underlying antidiabetic mechanisms. We first detail key extraction techniques and examine their metabolic fate, which is primarily characterized by gut microbiota-mediated deglycosylation leading to the formation of mogrol. Subsequently, the antidiabetic efficacy of mogroside-rich extracts and pivotal monomeric derivatives is critically evaluated, with an emphasis on mechanistic insights such as AMP-activated protein kinase (AMPK) pathway activation, anti-inflammatory and antioxidant activities, immunomodulatory effects, and the regulation of gut microbiota. It is important to note that due to the limitation of clinical trial data, most of the evidence reviewed derives from in vitro studies or animal models. Finally, their emerging role as functional ingredients within the food industry was discussed. Collectively, this review aimed to establish a robust scientific foundation for the development of mogrosides as safe, plant-derived sweeteners endowed with enhanced health-promoting properties for the prevention and management of diabetes.

## 1. Introduction

Mogrosides are cucurbitane-type triterpene glycosides that constitute the primary bioactive components of *Siraitia grosvenorii* (Luo Han Guo). They have garnered considerable interest owing to their unique sweetness, which reaches up to 300 times that of sucrose [1]. A systematic, quantitative bibliometric analysis of the existing academic studies on *Siraitia grosvenorii* extract and its primary sweetening components—mogrosides—as natural non-nutritive sweeteners further indicated that mogrosides have gained recognition as natural high-potency sweeteners [2]. Beyond sweetness, mogrosides exhibit multiple health-promoting activities, including antioxidant, anti-inflammatory, and antidiabetic effects [3]. Several countries and regions recognize *Siraitia grosvenorii* and its extracts as safe and list them among approved food additives. In the United States, *Siraitia grosvenorii* extract is classified as Generally Recognized as Safe (GRAS) by the Food and Drug Administration (FDA) [4]. Similarly, it is approved as an intense sweetener by Food Standards Australia New Zealand (FSANZ) [5] and included in Health Canada’s List of Permitted Sweeteners [6]. In China, its specific usage levels are further stipulated under the national food additive standard GB1886.77-2016 (Standards for the use of food additives) [7]. Accordingly, their most established and immediate application is as non-caloric sugar substitutes in foods and beverages. Consequently, mogroside extracts are being integrated into low-calorie food and beverage formulations as natural sweeteners that offer palatability without adding calories or spiking blood glucose.

In this context, the global challenge posed by diabetes, particularly type 2 diabetes mellitus (T_2_DM), brings mogrosides into sharper focus. T_2_DM is a complex metabolic disorder characterized by insulin resistance and impaired insulin secretion, with contributing factors including obesity, ectopic lipid accumulation, chronic inflammation, and dysregulated organ crosstalk [8]. The global diabetes burden continues to rise sharply. According to the IDF Diabetes Atlas (2025), approximately 589–590 million adults (≈11.1%) were living with diabetes in 2024, with projections reaching ~853 million by 2050 [9]. Moreover, a significant number of cases remain undiagnosed, highlighting critical gaps in early intervention [10]. In addition, the long-term burden of hyperglycemia encompasses microvascular (retinopathy, nephropathy, neuropathy) and macrovascular complications, with disproportionate effects in low- and middle-income countries [11]. Current management strategies for diabetes focus on individualized glycemic control and the reduction in cardiovascular and renal risks. This integrated approach commonly relies on combination pharmacotherapy, with key agents including metformin, glucagon-like peptide-1 (GLP-1) receptor agonists, sodium–glucose cotransporter 2 (SGLT2) inhibitors, and insulin [12]. However, these antidiabetic medications are often associated with adverse effects such as hypoglycemia, weight gain, gastrointestinal disturbances, and secondary failure [13]. These limitations highlight the need for complementary approaches that can support glycemic control with improved safety profiles.

Mogrosides emerge as a promising candidate for adjunctive dietary management of diabetes. They have demonstrated potential in modulating glucose metabolism through multiple pathways, including AMP-activated protein kinase (AMPK) activation and Takeda G protein-coupled receptor 5 (TGR5)-mediated insulin secretion [14,15]. Integrating these mechanisms with their inherent anti-inflammatory and antioxidant properties, mogrosides also suggest potential for both glycemic control and the prevention of diabetes-related complications [16]. However, the reported findings are supported predominantly by in vitro experiments and animal models, and their clinical relevance has not been confirmed by large-scale randomized controlled trials. Importantly, as a non-caloric sweetener, mogrosides offer a unique dual advantage for individuals with diabetes: they provide sweetness without raising blood glucose, while simultaneously offering potential metabolic benefits. Conceptually, mogrosides can be discussed in two distinct yet related contexts: as approved non-caloric sweeteners that replace added sugars in the diet, and as bioactive constituents for which metabolic benefits have been proposed based on experimental studies. Moreover, their established safety profile across multiple regulatory jurisdictions further supports their feasibility as a dietary adjunct.

**Figure 1:**
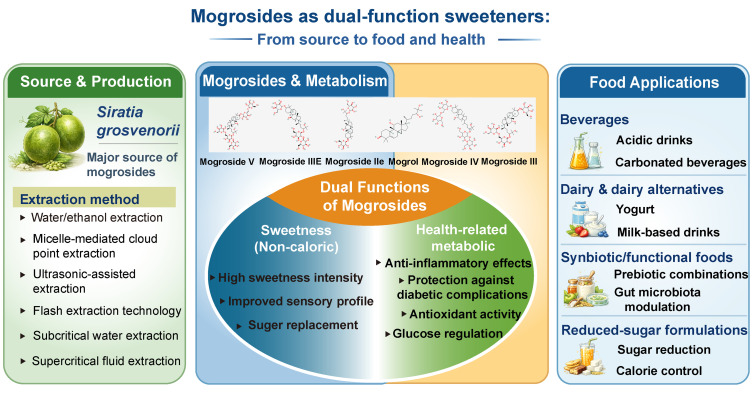
Mogrosides as dual-function sweeteners: From source to food and health (Chemical structure in the figure. Black indicates carbon atoms, red indicates oxygen atoms, and gray-blue indicates hydrogen atoms).

**Figure 2:**
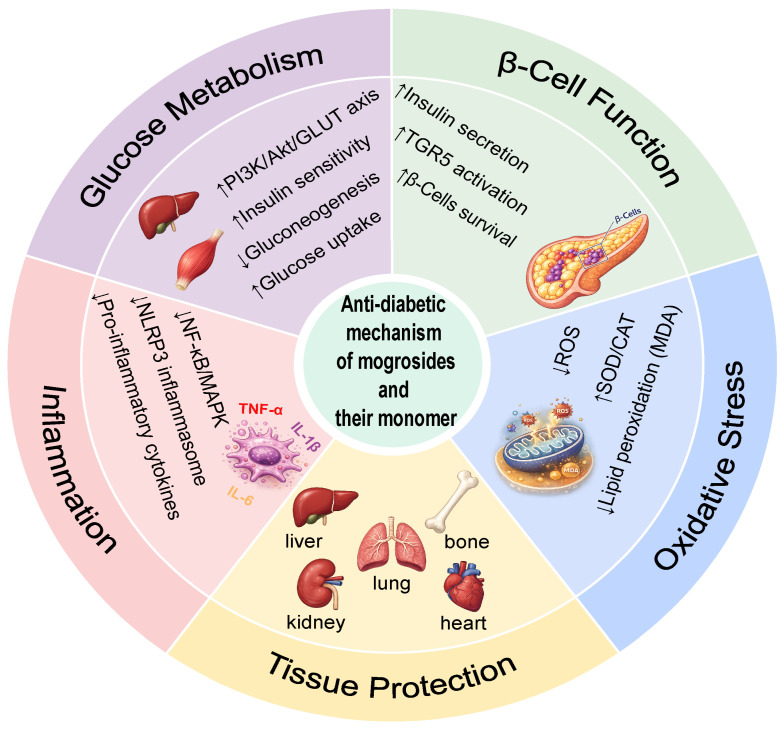
The anti-diabetic mechanism of mogrosides and their monomers.

To advance the practical application of *Siraitia grosvenorii* extracts and mogrosides, this review systematically outlines their extraction methods, metabolic activities, and current application within the food industry (Figure 1). It further evaluates the antidiabetic effects and underlying mechanisms of mogrosides and their monomeric components (Figure 2). Searches were conducted on PubMed and the Web of Science Core Collection, with the most recent search completed in February 2026. Search terms included *Siraitia grosvenorii* or monk fruit or luo han guo, mogroside or mogroside V or mogrol, as well as keywords related to extraction, metabolism/biotransformation, and anti-diabetic effects/mechanisms. Despite the scarcity of clinical research, these studies were prioritized whenever possible. Otherwise, in vivo studies were preferred for mechanistic support, followed by in vitro evidence. By integrating current evidence, this work aims to provide a scientific basis for the use of mogrosides as a safe, plant-based sweetener with added health benefits in diabetes management. By integrating current evidence, this work aims to provide a scientific basis for the use of mogrosides as a safe, plant-based sweetener with added health benefits in diabetes management.

## 2. The Extraction Methods of Mogrosides

Multiple extraction techniques have been developed to isolate mogrosides from *Siraitia grosvenorii*, each exhibiting distinct characteristics and operational parameters. These methods include micelle-mediated cloud-point extraction (CPE), ultrasonic-assisted extraction (UAE), flash extraction technology (FE), subcritical water extraction (SWE), and supercritical fluid extraction (SFE).

CPE is a liquid–liquid extraction technique that utilizes the unique phase-separation behavior of surfactant solutions at elevated temperatures to concentrate and isolate target analytes. When a nonionic surfactant is added to an aqueous sample above its critical micelle concentration, it forms micelles that encapsulate hydrophobic or complexed analytes. Upon heating to the cloud-point temperature, the solution separates into two phases: a surfactant-rich phase containing the micelle-entrapped analytes and a dilute aqueous phase. The analyses are thus extracted and concentrated into the surfactant-rich phase, which can then be collected and analyzed. For mogroside extraction, the CPE technique, which utilized nonionic surfactants such as Genapol X-080 (Sigma-Aldrich, St. Louis, MO, USA), demonstrated excellent efficiency. This method had achieved a high mogroside V yield of 80.7%. Furthermore, it offered a significant pre-concentration factor of 10.8 [17].

UAE employs high-frequency ultrasonic waves (typically 20–200 kHz) to improve the extraction efficiency of target compounds from various matrices. The mechanism is based on acoustic cavitation, where ultrasonic energy generates and implodes microscopic bubbles in the solvent, creating localized high-pressure and high-temperature microenvironments. This phenomenon effectively disrupts cellular structures, enhances solvent permeation, and accelerates mass transfer kinetics. A notable application of UAE involved the extraction of bioactive mogrosides from *Siraitia grosvenorii*, where optimized conditions (20 kHz, 50 °C, 40 min) achieved a remarkable yield of 3.97% with 91.84% purity [18]. Compared to traditional extraction methods, UAE offers distinct advantages, including superior extraction efficiency, reduced processing time, and lower solvent consumption. However, the translation of this technique to an industrial scale encounters a significant hurdle. A major barrier is the high initial investment needed for dedicated ultrasonic systems. Furthermore, scaling up the process presents technical difficulties, particularly in ensuring uniform and reproducible cavitation effects.

FE is an innovative method characterized by exceptional speed. It operates by combining intense mechanical shearing, at speeds ranging from 3000 to 30,000 rpm, with instantaneous solvent penetration. As a result, it achieves the efficient separation of target compounds in a matter of seconds. Its efficacy was exemplified by the successful extraction of mogrosides from *Siraitia grosvenorii*, which achieved an impressive yield of 4.11%. Notably, the process also preserved compound integrity while minimizing solvent consumption [19].

SWE is an innovative green extraction technique that exploits the tunable solvation properties of water under elevated temperature (100–374 °C) and pressure. This method leverages the dramatic reduction in water’s dielectric constant (from 70 to near 1) with increasing temperature, allowing selective extraction of compounds based on polarity. Research has demonstrated that optimal extraction of mogrosides with enhanced antioxidant activity can be achieved at 140 °C using a 15% ethanol co-solvent system for 20 min [20].

SFE represents an advanced separation technique. It utilizes a solvent in its supercritical state, which typically involves carbon dioxide (CO_2_) maintained above its critical temperature and pressure point. In this state, the fluid exhibits unique properties combining gas-like diffusivity and liquid-like density, enabling highly efficient penetration of matrices and selective compound dissolution. The solvation power of supercritical fluids is tunable through precise adjustments of pressure and temperature. This enables the selective extraction of compounds ranging from nonpolar to moderately polar. Furthermore, the system’s applicability can be broadened by introducing polar modifiers, such as ethanol. SFE utilizing carbon dioxide (CO_2_) modified with ethanol has shown potential for mogroside isolation. The addition of chromatographic supports, including silica or alumina, played a critical role in this process. This modification directly contributes to significantly higher recovery rates [21].

Overall, these diverse extraction methodologies each present a unique combination of yield, purity, scalability, environmental impact, and cost (Table 1). SFE with CO_2_ achieved the highest yield (83%), followed by CPE (80.7%) and SWE (70.5%). In contrast, SWE and FE yielded substantially lower recoveries (3.97% and 4.11%, respectively). Purity was only reported for UAE, which exhibited a high value of 91.84%. As for cost, CPE and SWE were rated as medium, SFE with CO_2_ as medium-to-low, and both UAE and FE as low. From an environmental perspective, the techniques differ considerably. SFE employs CO_2_, which is non-toxic and eliminates organic solvent use, offering a clean and environmentally friendly approach. SWE utilizes water as the solvent under subcritical conditions, presenting a green and sustainable alternative with minimal environmental burden. CPE relies on relatively small amounts of surfactants, reducing organic solvent consumption compared to conventional methods, though surfactant disposal requires consideration. UAE and FE are energy-intensive techniques. While they reduce solvent usage through enhanced mass transfer, their overall environmental footprint is influenced by electricity consumption and the potential need for subsequent purification steps. Regarding operational complexity: UAE and FE are relatively straightforward to operate, requiring standard laboratory equipment and minimal specialized training, making them accessible for routine applications. CPE involves moderate operational complexity due to the need for precise temperature control and phase separation steps, though it remains feasible for most laboratory settings. SWE requires specialized equipment capable of maintaining high temperature and pressure, demanding skilled operation and rigorous safety protocols. SFE is the most operationally demanding, requiring high-pressure systems, CO_2_ handling expertise, and sophisticated process control, which may limit its adoption in facilities lacking specialized infrastructure.

The selection of an optimal extraction method fundamentally depends on a comprehensive evaluation of multiple factors, including: (1) the physicochemical properties of the target compound (e.g., polarity, thermal stability), (2) the required purity and yield, (3) environmental considerations, and (4) infrastructural and economic constraints. Modern strategies for extraction development are increasingly shifting toward integrated approaches. These methods systematically combine multiple techniques to leverage their complementary advantages, aiming to mitigate the inherent limitations of any single method used in isolation.

## 3. The Metabolism of Mogrosides

Metabolic transformations play a pivotal role in modulating the pharmacological activity of bioactive compounds. This modulation occurs primarily through three fundamental mechanisms: (1) the metabolic activation of prodrugs [22], (2) the enzymatic inactivation of active compounds [23], and (3) the bioactivation to reactive or toxic metabolites [24].

As sweet cucurbitane-type triterpenoid glycosides, mogrosides exhibit low oral bioavailability (ranging from 3.5% to 10.3%), demonstrating a clear glycoside order effect: a longer sugar chain (e.g., mogroside V) confers higher water solubility but lower bioavailability, whereas a shorter sugar chain (e.g., Mogrol) increases lipophilicity and results in slightly higher bioavailability [25,26]. While, mogrosides undergo significant metabolism after ingestion that critically influences their tissue distribution, and ultimate biological effects [27]. Although Zhang et al. [28] reviewed mogroside V from the perspectives of structure, synthesis, pharmacokinetics, toxicity, and broad pharmacological activities, a comprehensive summary of the metabolism of other mogrosides is still lacking. Moreover, the extent to which these metabolic conversions translate into measurable systemic exposure (e.g., circulating mogrol and related metabolites) remains an important determinant of clinical relevance. To improve clarity, the pharmacokinetic-related findings currently available are summarized in Table 2.

The biotransformation process is driven by two major pathways: (1) enzymatic hydrolysis mediated by digestive enzymes (e.g., β-glucosidases), and (2) microbial metabolism by commensal gut microbiota. These metabolic reactions sequentially cleave glucose moieties from the parent compounds such as mogroside V, generating intermediate metabolites including mogroside IIIE, mogroside IIIA1, and siamenoside I. Finally, the complete removal of sugar moieties yields the aglycone core, mogrol [29]. For instance, in vitro studies using both male and female human fecal homogenates have demonstrated that mogrosides, including mogroside IIIE, siamenoside I, mogroside V, and isomogroside V, share a common metabolic fate: they are extensively metabolized into mogrol within 24 h under anaerobic conditions. This confirms deglycosylation as the primary metabolic pathway. Notably, at a concentration of 200 μg/mL, these compounds exhibited comparable deglycosylation rates, with near-complete disappearance of parent compounds and concurrent mogrol formation within 8 h. However, concentration-dependent metabolic differences emerged at 2000 μg/mL, where disappearance ratios varied significantly among compounds (siamenoside I: 87%; isomogroside V: 94%; mogroside V: 62%; mogroside IIIE: 35%) during the same incubation period, suggesting potential substrate inhibition or metabolic saturation effects [30]. In addition, Zhou et al. [31] investigated the metabolism of mogrosides extracted from *Siraitia fructus* by human intestinal microbiota from normal and T_2_DM samples. The study identified 12 and 19 metabolites in normal and T_2_DM microbiota groups, respectively, including 7-oxomogroside IV and mogroside IIe. The metabolic transformations involved multiple reactions: deglycosylation, oxidation, isomerization, and deoxidation. Notably, the T_2_DM group showed enhanced oxidative and isomerization activities, likely reflecting disease-associated alterations in microbial composition and enzymatic profiles. Moreover, interindividual variability in microbiota composition and functional capacity may also lead to substantial person-to-person differences in mogroside-to-mogrol conversion and metabolite patterns, thereby introducing uncertainty in predicting systemic exposure and efficacy across populations.

These in vitro findings are corroborated by in vivo studies, which provide further insight into the biotransformation of mogrosides under physiological and pathological conditions. In normal and T_2_DM rats, orally administered with mogroside V and related glycosides (1–3 glucose residues) at 200 mg/kg, exhibited deglycosylation to their metabolites, including mogroside IIIA1, mogroside IIA1, and mogroside IA1. There were differences in mogroside V and mogrosides metabolism between the normal and T_2_DM rats. Consistently, after oral mogroside V administration, mogroside IIIA1 showed significantly higher exposure in T_2_DM rats, with maximum plasma concentration (C_max_) = 163.80 ± 25.56 ng/mL and area under the curve (AUC_0-t_) = 2327.44 ± 474.63 h·ng/mL (*p* < 0.05), representing increases of ~59% versus controls. This was accompanied by downregulation of intestinal tight-junction marker zonula occludens-1 (ZO-1) in the duodenum and colon (*p* < 0.05), suggesting impaired barrier integrity [32]. A more comprehensive metabolic investigation by Yang et al. [33] examined mogroside III and IIIE (50 mg/kg) in normal versus drug-metabolizing enzyme-induced rats. In this study, a total of 76 to 121 metabolites were identified, suggesting both compounds underwent extensive biotransformation, including deglycosylation, dehydrogenation, hydroxylation, and oxidation. The primary metabolites included secondary glycosides (e.g., mogroside IIA, IIIA, IE1), the aglycone mogrol, and highly oxidized mogrol derivatives. Moreover, phenobarbital-induced enzyme activation substantially increased metabolite diversity and abundance, implicating cytochrome P450 enzymes as major contributors to mogroside metabolism [34]. This highlights the potential for drug–diet interactions or host metabolic status to shift metabolite profiles and systemic exposure, further complicating translational extrapolation. In a related context, a 12-month randomized trial in adults with obesity showed that ketogenic and time-restricted dietary interventions improved adiposity and glycemic indices in parallel with increased circulating Orexin-A, which was associated with metabolic improvements [35]. Further, metabolite profiling of siamenoside I in rats also revealed extensive metabolic pathways involving deglycosylation, hydroxylation, dehydrogenation, deoxygenation, and isomerization reactions. Notably, siamenoside I metabolites exhibited differential tissue distribution, predominantly accumulating in the intestine (19), stomach (21), kidney (13), and brain (14), with mogroside IIIE being the most widely distributed metabolite [36]. It should be noted that such pharmacokinetic studies commonly employ doses that are substantially higher than exposures expected from typical use levels in functional foods, making direct translation of plasma concentrations to dietary settings uncertain.

In summary, mogrosides are predominantly deglycosylated by intestinal enzymes and gut microbiota, generating intermediate glycosides and ultimately the aglycone mogrol. Their pharmacokinetic behavior and metabolite profiles can be reshaped by host factors, as evidenced by altered biotransformation under diabetic conditions and following induction of drug-metabolizing enzymes. In addition, although mogrol and related metabolites are plausible mediators of bioactivity, translational interpretation remains cautious due to limited human pharmacokinetics data, microbiota-driven interindividual variability, and the difficulty of extrapolating exposure from high-dose animal studies to typical dietary-use scenarios.

## 4. The Anti-Diabetic Effects of *Siraitia grosvenorii* Extract, Mainly Mogrosides

T_2_DM is a chronic metabolic disorder marked by persistent hyperglycemia and disturbed lipid metabolism. Its harm extends far beyond abnormal blood glucose levels, which also damages blood vessels and nerves, leading to devastating complications, including heart attacks and strokes [37], diabetic kidney disease, retinopathy, and neuropathy [38]. Moreover, gestational diabetes will affect the health of future generations [39]. As such, diabetes has emerged as the top chronic disease threatening global longevity and quality of life. The management of diabetes often includes synthetic drugs. However, these treatments can have adverse side effects. As such, natural products, especially those derived from plants like *Siraitia grosvenorii*, have become a focus of research due to their efficacy and lower toxicity profiles. It should be noted that plant-derived ingredients are increasingly explored as dietary adjuncts that may complement standard care rather than replace evidence-based pharmacotherapy. Furthermore, a clear distinction still needs to be made between the pharmacological doses commonly used in animal studies and the realistic exposure levels achievable through dietary intake.

*Siraitia grosvenorii* extract, primarily composed of mogrosides, has demonstrated anti-hyperglycemic and anti-hyperlipidemic effects in experimental models, positioning it as a promising nutraceutical and therapeutic agent for diabetes and metabolic syndrome management. These unique cucurbitane-type triterpenoid glycosides exhibit a broad spectrum of pharmacological activities, including anti-inflammatory, anti-cancer, and antioxidative properties. Recent studies have shown that the mogrosides extract from *Siraitia grosvenorii* has significant potential in alleviating diabetes and its complications. For example, mogrosides-rich extracts have demonstrated considerable effects in reducing fasting blood glucose, serum insulin levels, and insulin resistance in both type 1 and type 2 diabetic animal models [40,41]. The mechanisms behind these effects are linked to the attenuation of oxidative stress and the activation of the AMPK pathway. AMPK activation plays a critical role in improving insulin sensitivity. It reduces hepatic glucose production and promotes fat oxidation, helping address both hyperglycemia and hyperlipidemia [42].

Oxidative stress, exacerbated by hyperglycemia, is a significant contributor to the pathogenesis of diabetic complications, including nephropathy and retinopathy. Mogrosides possess potent antioxidant properties, scavenging free radicals and restoring the antioxidant defense system. This has been shown in both in vitro and in vivo models, where treatment with mogrosides decreased malondialdehyde (MDA) levels and increased the activity of antioxidants like superoxide dismutase (SOD) and glutathione peroxidase (GSH-Px) [43].

In addition to their effects on glucose and lipid metabolism, mogrosides also modulate the immune system. In diabetes, an imbalance in T-helper cells (Th1/Th2) contributes to immune dysfunction and exacerbates disease progression. Mogrosides have been shown to shift this imbalance towards a more beneficial Th2 response, which helped to mitigate inflammatory damage to pancreatic islet cells, offering further protection against diabetes progression [44].

Recent research has also indicated that mogrosides exert their antidiabetic effects through the modulation of gut microbiota. In T_2_DM rats, treatment with *Siraitia grosvenorii* extract and low-polar mogroside fractions restored gut microbiota composition, increasing beneficial short-chain fatty acids (SCFAs) and promoting insulin sensitivity [45]. This highlights a novel mechanism in which mogrosides influence the gut microbiota to enhance their antidiabetic effects. However, microbiota responses are sensitive to baseline community structure, diet composition, and study duration, and these factors vary substantially across animal experiments, potentially contributing to inconsistent effect sizes and limiting generalizability to humans.

In addition, mogrosides not only reduce blood glucose but also improve lipid profiles, which are crucial in preventing cardiovascular complications commonly associated with diabetes [40,46]. The modulation of oxidative stress, insulin sensitivity, and immune function positions mogrosides as a multi-target therapeutic agent [47]. Furthermore, the protective effects on renal and hepatic functions suggested a significant role in mitigating diabetic nephropathy and hepatosteatosis [48,49].

Overall, mogrosides from *Siraitia grosvenorii* represent a promising natural therapeutic candidate for the management of diabetes and its complications. Their mechanisms of action, including the modulation of oxidative stress, immune function, and gut microbiota, underscore their multifaceted role in glycemic control (Figure 2). However, many of the cited studies employ doses that are not achievable through normal dietary intake, and several methodological limitations remain, including short intervention durations, small sample sizes, and the absence of human validation.

## 5. The Anti-Diabetic Effect of Mogroside Monomer

While studies on *Siraitia grosvenorii* extracts provide compelling evidence for the collective anti-diabetic potential of mogrosides, the distinct contributions of individual mogroside monomers warrant detailed examination. The bioactivity of the extract results from the combined and possibly synergistic effects of its constituent glycosides, each with unique structural features, pharmacokinetic properties, and mechanistic pathways. Investigating these monomers separately allows for a clearer understanding of structure-activity relationships, precise molecular targets, and optimal therapeutic configurations. Therefore, the following sections focus on key mogroside monomers: Mogroside V (MO5), mogroside IIIE (MG IIIE), mogroside IIe (MGE IIe), mogrol, and minor mogrosides. These compounds play specific roles in glucose homeostasis, insulin signaling, inflammation, oxidative stress, and complication management. Table 3 summarizes the anti-diabetic effects of mogrosides and their monomers.

### 5.1. MO5

MO5 showed excellent antidiabetic potential spanning pancreatic, hepatic, immune, and complication-related pathways. Experimental evidence from animal models has supported these broad mechanisms. In a direct comparison of different sweeteners in T_2_DM mice models, MO5 regulated systemic blood glucose and lipid levels while improving protein metabolism markers. At the molecular level, MO5 exerted its effects through several key mechanisms. It reduced inflammation by inhibiting the NLR Family, Pyrin domain containing 3 (NLRP3) inflammasome, which plays a central role in insulin resistance and the development of diabetes-related complications such as nephropathy and cardiomyopathy [52]. Furthermore, research has shown that MO5 played a crucial role in the regulation of glucose metabolism by modulating the Phosphatidylinositol 3-kinase (PI3K)/Protein kinase B (PKB)/Glucose transporter type 2 (GLUT2) axis, which is vital for improving insulin sensitivity and glucose uptake [62]. Beyond systemic and molecular actions, MO5 also influenced gut-mediated pathways. An accompanying study observed its beneficial effects on intestinal barrier function and gut microbiota, suggesting that the gut axis may serve as a key regulatory point for systemic insulin sensitivity [63,64]. Additionally, MO5 exhibited antioxidant properties, helping to protect against oxidative stress-induced damage, which contributed to the prevention of diabetes complications such as nephropathy and retinopathy [16]. These mechanistic insights are complemented by its therapeutic potential against specific diabetic complications. For instance, in hepatic steatosis, MO5 improved metabolic profiles by activating the AMPK pathway and protecting the liver from fatty liver disease, which is directly related to enhanced insulin sensitivity at the liver level [48]. Moreover, MO5 has demonstrated therapeutic benefits in the repair of alveolar bone defects in diabetic rats, suggesting its potential role in tissue regeneration and wound healing under diabetic conditions [50]. Parallel to these pharmacological findings, significant progress has been achieved in understanding the production of MO5. Recent studies have elucidated its biosynthetic pathways in *Siraitia grosvenorii*. A novel transcription factor, SgTCP24, plays a key role by activating enzymes such as SgSQE, SgCS, and SgCYP87D18, thereby enhancing MO5 synthesis [65]. Advances in synthetic biology have enabled the de novo production of MO5 in engineered yeast systems, providing scalable production alternatives to traditional plant-based extraction [66]. Additionally, improvements in UDP-glycosyltransferase engineering have enhanced the conversion of precursors to MO5, expanding its commercial potential [67]. These developments highlighted significant progress in metabolic engineering and synthetic biology for MO5 production.

In summary, these findings collectively support the use of MO5 as a potential adjunctive therapy for diabetes management, highlighting its multifaceted effects on glucose metabolism, insulin sensitivity, and diabetic complications (Figure 2). However, further clinical trials are necessary to validate these effects in human populations, particularly focusing on the optimal dosage, safety, and potential interactions with other antidiabetic medications. Moreover, pharmacokinetic studies indicated that MO5 is metabolized in vivo, with distribution and metabolism influenced by both healthy and diabetic states. Comparative studies in healthy versus T_2_DM rats also showed that disease status altered the metabolism and disposition of MO5 [68]. These findings suggested that while MO5 shows promising therapeutic effects, further research is needed to track its metabolic pathways and metabolites [69].

### 5.2. MG IIIE

MG IIIE exhibited therapeutic potential across inflammatory processes, oxidative stress, and metabolic dysfunctions associated with T_2_DM. Evidence from preclinical models underscores its role in glucose metabolism and diabetic complications. For instance, in rodent models, MG IIIE has been shown to reduce inflammation, oxidative stress, and apoptosis in podocytes under high glucose conditions, suggesting its potential for protecting against diabetic nephropathy [55]. In models of gestational diabetes mellitus, MG IIIE was shown to alleviate hyperglycemia and improve insulin sensitivity through AMPK activation [54]. Together, these findings highlighted the capacity of MG IIIE to modulate pivotal pathways in glucose homeostasis, positioning it as a promising adjunct in T_2_DM management.

In addition to its effects on glucose metabolism, MG IIIE’s anti-inflammatory properties are also significant in the context of diabetes. Chronic inflammation is a hallmark of T_2_DM and contributes to insulin resistance and related complications. Research has shown that MG IIIE can modulate immune responses by influencing the Toll-like receptor 4 (TLR4)/Mitogen-activated protein kinase (MAPK)/Nuclear factor-kappa B (NF-κB) axis, the pathways involved in inflammatory responses [70]. Furthermore, its antifibrotic effects, particularly through the TLR4 pathway, may be beneficial in preventing fibrosis-related complications, such as diabetic nephropathy and cardiovascular fibrosis [71]. However, the interpretation of the in vivo efficacy of MG IIIE should take its metabolic fate into account. Pharmacokinetic studies in normal and drug-metabolizing enzyme-induced rats indicated that MG IIIE undergoes extensive biotransformation, generating multiple metabolites via deglycosylation and oxidation. Enzyme induction markedly alters metabolite profiles and systemic exposure, suggesting that both the parent compound and its metabolites may contribute to biological activity and influence effective bioavailability [33].

In summary, mogroside IIIE demonstrated significant antidiabetic potential through multiple mechanisms, including enhancing insulin sensitivity via AMPK activation, reducing inflammation through TLR4 signaling, and protecting against oxidative stress and apoptosis (Figure 2). Although mogroside IIIE could be a promising adjunct therapy for diabetes, further clinical studies are needed to fully elucidate its efficacy and safety in human populations.

### 5.3. MGE IIe

MGE IIe demonstrated cardiometabolic benefits in diabetic contexts. In high-fat diet + streptozotocin induced T_2_DM and rat heart myoblast (H9c2) cells, MGE IIe lowered fasting glucose and atherogenic lipids, suppressed inflammatory cytokines, and attenuated myocardial apoptosis. These effects were mediated through the down-regulation of caspases-3/8/9/12, Bcl-2-associated X protein (Bax), and Cytochrome c (Cyt-c), alongside up-regulation of Bcl-2 [56]. Beyond cardiac-specific effects, MGE IIe also shows indirect but complication-relevant activity in other tissue injury models. For example, in models of acute pancreatitis, it alleviated pathological damage by modulating the Interleukin-9 (IL-9)/IL-9 receptor axis, while in acute lung injury, it exerted protective effects by disrupting the Secretory phospholipase A2 (sPLA2)–Group IIA (IIA)–Epidermal growth factor receptor (EGFR) signaling pathway and subsequent PKB/Mechanistic target of rapamycin (mTOR) activation [72,73]. Although these models are not inherently diabetic, the inflammatory and cytotoxic pathways targeted by MGE IIe are often amplified in diabetic conditions, making these mechanisms potentially relevant for protecting tissues vulnerable to diabetic complications.

### 5.4. Mogrol

Mogrol, the aglycone derivative of mogrosides, exhibits significant antidiabetic activity. In pancreatic β-cells, Mogrol acted as a TGR5 agonist, enhancing glucose-stimulated insulin secretion under normoglycemic conditions [15]. In KKAy diabetic mice, mogrol effectively lowered hyperglycemia mainly by boosting insulin release without increasing pancreatic insulin content or β-cell mass [74].

In addition to promoting insulin secretion, mogrol also modulated adipocyte metabolism. Studies in 3T3-L1 adipocytes demonstrated that mogrol suppressed adipogenesis and triglyceride accumulation via early inhibition of cAMP response element-binding protein (CREB) signaling and sustained AMPK activation, collectively enhancing insulin sensitivity [57,75]. Additionally, mogrol displays broader anti-inflammatory and pathway-modulating effects relevant to diabetic complications. In inflammation-rich models such as DSS-induced colitis, mogrol activated AMPK and Sirtuin 1 (SIRT1) while restraining NLRP3-driven cytokine signaling [76,77]. Although these pathways are not inherently diabetic, the mechanisms they involve, notably AMPK and NLRP3 inflammasome activation, are frequently dysregulated in diabetes. Such dysregulation contributes to both micro- and macrovascular injury. This connection, therefore, extends the mechanistic relevance of mogrol to diabetic tissue damage (Figure 2).

Looking forward, there are still several key research directions that remain to be addressed. Future studies should establish exposure–response relationships, confirm direct engagement of TGR5 in β-cells, compare the efficacy of mogrol with individual mogrosides in comparable diabetic models, and evaluate whether its AMPK-centered anti-inflammatory signaling confers organ protection in diabetic complications.

### 5.5. Other Minor Mogrosides (Mogroside IV, Siamenoside I, Mogroside III)

Other minor mogrosides, such as Mogroside III, Mogroside IV, and Siamenoside I, exhibited inhibitory effects on intestinal α-glucosidase and related disaccharidases, thereby delaying carbohydrate digestion and reducing postprandial glucose elevation [78]. Further enzyme-guided fractionation has demonstrated potent α-glucosidase inhibitory activity of several cucurbitane-type triterpenoids from *Siraitia grosvenorii*, with certain isolates showing in vitro efficacy comparable to or exceeding that of acarbose under standardized assay conditions, supporting the rationale for targeting postprandial hyperglycemia with these minor mogrosides. However, direct in vivo evidence for the individual bioactivities of Mogroside III, Mogroside IV, or Siamenoside I remains limited. There is also a notable absence of studies examining their effects on parameters such as serum uric acid or blood pressure in established animal or clinical models. Moving forward, key research priorities should include establishing exposure–response relationships and pharmacokinetic profiles for each monomer, verifying oral stability, and evaluating their efficacy in well-characterized glycemic and complication models. Such investigations will clarify how these minor mogrosides complement the effects of major compounds like Mogroside V, Mogroside IIIE, Mogroside IIe, and mogrol. Their complementary roles may span key processes such as insulin secretion, insulin sensitivity, oxidative stress, and inflammatory pathways. Ultimately, this will help build a more complete understanding of mogroside-based therapeutics.

However, current evidence on the antidiabetic activity of mogrosides and their individual monomers is largely derived from in vitro studies and rodent models. Well-powered human trials assessing efficacy using clinically relevant endpoints remain limited or absent. Furthermore, many animal studies employ relatively high doses and short intervention periods with small sample sizes, limiting their direct applicability to realistic dietary use. Critical aspects such as potential toxicity, long-term safety, and drug interactions have also not been systematically evaluated. These limitations represent fundamental gaps that must be addressed before any therapeutic application can be considered.

## 6. Anti-Diabetic Efficacy of Mogrosides Combined with Other Substances

Building on the anti-diabetic effects of mogrosides, particularly in modulating insulin sensitivity, glucose metabolism, and inflammation, it is crucial to explore their potential synergistic effects when combined with other medications. Current combination evidence primarily comes from nutraceutical-style co-formulations and multi-extract studies that include mogrosides (Table 4). For example, a triple beverage formulation containing stevia glycoside, Mogroside V, and sodium hyaluronate (each at 0.1 mg/mL) improved both glycemia and profiles, and outperformed any single ingredient across in vitro and in vivo studies [79]. This suggested genuine functional complementarity beyond mere sweetness substitution. Similarly, in vitro and in silico screening studies of *Siraitia grosvenorii* with *Dimocarpus longan* and *Orthosiphon stamineus* showed synergistic or supra-additive effects across α-amylase, α-glucosidase, dipeptidyl peptidase-4 (DPP-4), glucose uptake, and antioxidant assays, with network pharmacology and docking converging on carbohydrate-digestion and incretin-relevant targets [80,81].

Despite these promising leads, important limitations remain. To date, there have been no animal or clinical trials that have tested a purified mogroside monomer in combination with common antidiabetic drugs such as metformin, DPP-4 inhibitors, or SGLT2 inhibitors. Moreover, key pharmacokinetic and pharmacodynamic parameters, including purity, stability, systemic exposure, and target engagement of individual mogrosides in humans, remain incompletely characterized.

From a mechanistic perspective, potential pharmacokinetic interactions may arise through two distinct pathways. First, mogrosides undergo extensive gut microbiota-mediated deglycosylation to yield bioactive metabolites such as mogrol. Consequently, any co-administered drug that alters microbial composition or intestinal physiology could plausibly affect mogroside conversion efficiency and systemic metabolite exposure. Second, at the pharmacodynamic level, mogrosides activate AMPK-a central node in glucose and energy metabolism that is also targeted by metformin-raising the possibility of additive or synergistic effects when used concurrently. However, whether such convergence translates into clinically meaningful interactions remains unknown in the absence of human data.

Given these uncertainties, potential drug-mogroside interactions warrant careful prospective monitoring. For instance, combining mogrosides with α-glucosidase inhibitors may increase gastrointestinal side effects, as has been observed with other digestive enzyme inhibitors [83]. Similarly, the impact of mogrosides on incretin signaling dynamics-particularly in combination with DPP-4 inhibitors-warrants further investigation, as such combinations could potentially alter insulin secretion pathways [57]. Likewise, concurrent use with metformin may result in combined metabolic effects through shared AMPK pathway activation [84]. Future studies also should specifically quantify both metabolite exposure and microbiota-associated variability to clarify whether meaningful pharmacokinetic interactions occur in practice, and should distinguish between pharmacodynamic convergence (e.g., AMPK) and pharmacokinetic alterations (e.g., microbiota-mediated deglycosylation) when assessing combination strategies. Addressing these limitations through structured preclinical and clinical studies will be essential to substantiate the therapeutic potential of mogrosides as adjuncts to conventional antidiabetic regimens.

## 7. The Application of Mogrosides in the Food Industry

Mogrosides exhibit a favorable safety profile. In Japan, China, and United States, *Siraitia grosvenorii* extract (mainly including Mogrosides) have been approved as dietary supplements [40,69,85]. Thus, they have emerged as a promising natural sweetener, not only as a sugar substitute but also as a functional ingredient. This makes them particularly attractive for incorporation into functional foods targeting diabetic and health-conscious consumers. As shown in Table 5, a notable application involves the use of mogroside V as a natural sweetener in synbiotic yogurts. Studies in T_2_DM rats have demonstrated that such yogurts improve glucose regulation, lipid metabolism, and gut microbiota composition. These benefits are linked to the activation of the hepatic AMPK signaling pathway [86]. Moreover, the anti-inflammatory, antioxidant, and anti-obesity effects of mogrosides further support their role in functional foods targeting metabolic health [87]. For instance, incorporating mogrosides into baked goods and beverages not only reduces sugar content but also enhances the antioxidant capacity of these products, contributing to improved health outcomes [88]. For clinical research, in healthy males, a *Siraitia grosvenorii* extract-containing beverage increased lunch intake without affecting total daily intake or 24 h glycemic parameters versus sucrose, while a mogroside V/stevia blend reduced 2 h insulin incremental area under the curve in adults with overweight/obesity [89,90,91].

## 8. The Challenge of Adding Mogrosides to Food

The successful integration of mogrosides into food products relies heavily on managing their stability, flavor profile, and texture. Mogroside V demonstrates optimal stability in acidic beverages, especially around pH 3, which is typical for carbonated drinks and fruit juices. However, under conditions of extreme acidity, its stability can be compromised. Therefore, appropriate buffering may be required to prevent degradation in such environments [26]. Furthermore, recent studies indicated that mogrosides retained their sweetening properties even under thermal processing, supporting their suitability for a variety of food applications [95]. Ongoing research on the effects of light exposure and heat treatment remains essential for expanding their use across different food systems.

Beyond stability, flavor optimization is another critical aspect. While mogrosides provide high-intense sweetness, they are often accompanied by a lingering aftertaste. To address this, enzymatic transglycosylation has been proposed, which not only reduces undesirable aftertastes but also smoothens the sweetness profile, thereby enhancing consumer acceptability and overall flavor experience [96]. Building on this improvement, the modified mogroside V can be combined with galacto-oligosaccharides (mMV-GOS). This approach preserved sweetness while also exhibiting prebiotic activity in human fecal fermentation models, offering a promising strategy for formulating foods that support gut health [97]. Additionally, blending mogrosides with other sweeteners such as erythritol or stevia has been shown to further mitigate aftertaste and improve the sensory quality of final products [98]. In addition, beyond their most immediate and well-supported application as non-caloric sweeteners for sugar reduction, mogrosides have been proposed to exert antidiabetic and other physiological effects. This may be helpful for regulatory approval of mogrosides as a food ingredient. However, such claims remain predominantly supported by in vitro and animal studies. Further clinical research is necessary to verify their relevance in humans. On the contrary, the presence of additional biological effects at the cellular and tissue level raises legitimate questions regarding possible adverse outcomes and long-term health impacts. Even if such effects appear favorable in preliminary studies, they may introduce unforeseen risks under chronic exposure or in specific populations. Therefore, future development of mogrosides as sweeteners with purported secondary benefits must be accompanied by rigorous human validation, exposure-relevant toxicological assessment, and long-term safety evaluation.

## 9. Conclusions and Future Perspective

This review systematically synthesizes evidence on mogrosides, highlighting their dual role as natural, non-caloric sweeteners and promising adjunct for diabetes management. Key findings demonstrated that optimized extraction methods can efficiently isolate mogrosides. Following ingestion, they are primarily metabolized in the gut to bioactive compounds like mogrol. The antidiabetic potential of both extracts and key monomers (e.g., Mogroside V, IIIE) is well-supported in preclinical models. However, human interventional evidence remains limited, underscoring the need for larger randomized controlled trials. Their mechanisms are multifaceted, involving the improvement of insulin sensitivity, enhancement of insulin secretion, inhibition of carbohydrate-digesting enzymes, modulation of gut microbiota, and reduction in inflammation and oxidative stress. These actions contribute to improved glycemic/lipid control and mitigation of complications.

Moreover, to realize mogrosides’ potential, future efforts must focus on: (1) clinical validation through rigorous human trials; (2) mechanistic insight into molecular targets and monomer-specific effects; (3) synergistic therapies with conventional drugs; (4) sustainable production via advanced extraction and biotechnological synthesis, and (5) advanced applications in next-generation functional foods. Addressing these priorities will establish mogrosides as an evidence-based component of diabetes care and metabolic health.

## Figures and Tables

**Table 1:** Comparative summary of mogroside extraction techniques.

Extraction Technique	Yield	Purity	Scalability	Cost-Effectiveness	Environmental Impact
Micelle-mediated cloud-point extraction	80.7%	NR	Medium	Medium	Low–Medium
Ultrasonic-assisted extraction	3.97%	91.84%	Medium–Low	Medium–Low	Medium
Flash extraction technology	4.11%	NR	Medium	Medium–Low	Medium
Subcritical water extraction	70.5%	NR	Medium	Medium	Low–Medium
Supercritical fluid extraction (CO_2_ + modifier)	83%	NR	Medium	Low	Low–Medium

NR, not reported.

**Table 2:** The summary of the metabolism of mogrosides.

Main Compounds	Model	Dose	Key Results	Quantitative Parameters
Mogroside V, mogroside IIIE, siamenoside I, isomogroside V	Human fecal homogenates (in vitro, anaerobic)	200 μg/mL	Extensive deglycosylation to mogrol	Near-complete disappearance of parent compounds and concurrent mogrol formation within 8 h
Mogroside V, mogroside IIIE, siamenoside I, isomogroside V	Human fecal homogenates (in vitro, anaerobic)	2000 μg/mL	Concentration-dependent metabolic differences	Disappearance at 8 h: siamenoside I 87%, isomogroside V 94%, mogroside V 62%, mogroside IIIE 35%
Mogrosides extracted from *Siraitia fructus*	Human intestinal microbiota from normal vs. type 2 diabetes mellitus (T_2_DM) donors	Not reported	Metabolism differed by donor metabolic status	Metabolites identified: 12 (normal) vs. 19 (T_2_DM)
Mogroside V and related glycosides	Normal and T_2_DM rats (in vivo)	200 mg/kg, (oral)	Deglycosylation to mogroside IIIA1, IIA1, and IA1	Mogroside IIIA1 in T_2_DM rats: C_max_ = 163.80 ± 25.56 ng/mL; AUC_0-t_ = 2327.44 ± 474.63 h·ng/mL, ~59% increase vs. controls
Mogroside III and mogroside IIIE	Normal vs. drug-metabolizing enzyme-induced rats (in vivo)	50 mg/kg	Extensive biotransformation including deglycosylation, dehydrogenation, hydroxylation, and oxidation	Total metabolites identified: 76–121
Siamenoside I metabolites	Rat tissue distribution study (in vivo)	50 mg/kg	Metabolites distributed across multiple tissues. Mogroside IIIE was the most widely distributed	Metabolites detected: stomach 21, intestine 19, brain 14, kidney 13

T_2_DM, type 2 diabetes mellitus.

**Table 3:** Effects of Mogrosides and their monomers on diabetes and its complications.

Main Compounds	Model	Methods	Main Results	References
Mogroside V (MO5)	Diabetic rats with an alveolar bone defect	Implant nanofiber membrane containing 2% MO5; Assessed at 4 and 8 weeks	Promoted bone repair and tissue regeneration under diabetic conditions.	[50]
MO5	Diabetic mouse; Bone marrow mesenchymal stem cells (BMSCs)	Mice were fed with a high-fat diet (HFD) (60% fat, 20% protein, 20% carbohydrate) for 3 weeks, followed by streptozotocin injection. BMSCs were treated with MO5 at 6.25 mg/L for 24 h	Counteracted the osteogenic impairment; Restored mineralization; Upregulated miR-10b-5p; Activated the Phosphatidylinositol 3-kinase (PI3K)/Protein kinase B (PKB) signaling pathway.	[51]
MO5	Adult mouse hypothalamus neuronal cells	Treated with MO5 at 15/60/160 μM for 24 h	Altered up to 103 genes; Enriched AMP-activated protein kinase (AMPK), Forkhead box O (FoxO), and Notch pathways.	[14]
MO5	Polycystic Ovary Syndrome (PCOS) rat; Human ovarian granulosa cells (KGN)	PCOS rats received MO5 by gavage at 600 mg/kg for 30 d; KGN cells were treated with MO5 (50 nmol/mL) for 24 h	Inhibited NLR family, pyrin domain containing 3 (NLRP3) inflammasome activation; Reduced granulosa cell pyroptosis; Alleviated insulin resistance.	[52]
MO5	HFD-induced mice; LO2 hepatocytes	Mice received MO5 (25/50/100 mg/kg) by gavage for 8 weeks; LO2 cells were treated with MO5 at 15/30/60/120 μM for 24 h	Activated AMPK; Reduced hepatic lipid accumulation; Ameliorated steatosis linked to insulin resistance.	[48]
MO5	In vitro enzyme assay	α-glucosidase inhibition assay	Inhibited α-glucosidase activity with a Ki of 46.11 μM.	[53]
Mogroside IIIE (MG IIIE)	Gestational diabetic mice	Mice received 20 mg/kg MG IIIE for 16 d	Reduced fasting blood glucose and insulin levels; Improved glucose and insulin tolerance; Improved fetal outcomes; Suppressed inflammatory cytokines.	[54]
MG IIIE	High glucose-induced podocyte	Treated with MG IIIE at 1/10/50 μM for 24 h	Increased cell viability; Reduced the levels of inflammatory cytokines and malondialdehyde; Enhanced antioxidant enzyme activities; Attenuated apoptosis.	[55]
Mogroside IIe (MGE IIe)	T_2_DM rat; H9c2 cardiomyocytes	Rats received MGE IIe at 30/60 mg/kg for 8 weeks; H9c2 cells were pretreated with 20/50/100 μM for 4 h	Reduced cardiomyocyte apoptosis; Improved heart function; Alleviated T_2_DM-induced cardiomyopathy by regulating apoptosis-related proteins.	[56]
Mogrol	Pancreatic β-cells; Diabetic mice	Mice were treated with an HFD containing 0.01% or 0.05% mogrol for 5 weeks. INS-1 β-cells were treated with 50 μM mogrol for 24 h	Activated G-protein-coupled bile acid receptor 1; Increased insulin secretion; Raised plasma insulin; Improved glucose tolerance.	[15]
Mogrol	3T3-L1 preadipocytes	Pretreated with 20 μM mogrol for 30 min	Inhibited adipocyte differentiation; Reduced lipid accumulation; Suppressed cAMP response element-binding protein phosphorylation; Activated AMPK.	[57]
Mogroside derivatives	Hepatocellular Carcinoma cells (HepG2); T_2_DM rats	HepG2 cells were treated with mogrosides at 1/5/10 μM for 24 h; T_2_DM rats received MO5 at 30/75/150 mg/kg for 5 weeks	Reduced blood glucose levels in HepG2 cells; Improved insulin sensitivity in T_2_DM rats.	[41]
Mogrosides extract (MGE)	Alloxan-induced diabetic mice	Treated with 100/300/500 mg/kg MGE for 4 weeks	Reduced oxidative stress, serum glucose, and lipid levels in diabetic mice.	[40]
MGE	HFD-induced obese mice	Treated with 300 or 600 mg/kg MGE for 18 weeks	Improved obesity-related metabolic dysfunction by modulating gut microbiota; Reduced body weight; Improved glucose and lipid metabolism.	[58]
Mogrosides	α-glucosidase inhibition assay	α-glucosidase inhibition assays	Inhibited α-glucosidase activity.	[59]
*Siraitia grosvenorii* extract (SG-ex)	Obese T_2_DM rats	Treated with low-polar *Siraitia grosvenorii* glycosides at 0.020 g/kg for 14 d	Improved blood glucose and lipid levels.	[60]
SG-ex	T_2_DM Goto-Kakizaki rats	Diet supplemented with 0.4% SG-ex for 13 weeks	Improved oral glucose tolerance tests outcomes, with a higher early-phase insulin response; Increased pancreatic insulin content without affecting food intake or body weight.	[61]

MO5, mogroside V; BMSCs, bone marrow mesenchymal stem cells; HFD, high-fat diet; PI3K, Phosphatidylinositol 3-kinase; PKB, Protein kinase B; AMPK, AMP-activated protein kinase; FoxO, Forkhead box O; PCOS, polycystic ovary syndrome; KGN, human ovarian granulosa cells; NLRP3, NLR family, pyrin domain containing 3; MG IIIE, mogroside IIIE; MGE IIe, mogroside IIe; T_2_DM, type 2 diabetes mellitus; HepG2, hepatocellular carcinoma cells; MGE, mogrosides extract; SG-ex, *Siraitia grosvenorii* extract.

**Table 4:** Combination effects of Mogrosides or their monomers with other drugs on diabetes alleviation.

Main Compounds	Model	Methods	Main Results	References
Stevia glycoside (SG), Mogroside V (MO5), Sodium hyaluronate (SH)	Diabetic zebrafish	A mixture containing SH, SG, and MO5 (each at 0.1 mg/mL) was administered to zebrafish via immersion for 12 h per day over a period of 14 d	Demonstrated enhanced efficacy in glyco-lipid control and multiple complication-related parameters, superior to any single-ingredient monotherapy, while also delivering a sweetening effect that did not raise blood glucose.	[79]
*Siraitia grosvenorii*, *Dimocarpus longan*, *Orthosiphon stamineus*	In vitro; in silico	α-glucosidase and dipeptidyl peptidase-4 (DPP-4) inhibition assay; Network pharmacology analysis	Exhibited synergistic effects on glucose metabolism; Inhibited key enzymes involved in postprandial glucose regulation.	[80]
*Siraitia grosvenorii*, *Dimocarpus longan*, *Orthosiphon stamineus*	In vitro	α-amylase and α-glucosidase; DPP-4 inhibition assays; Glucose uptake tests; Antioxidant assays	Multi-extract combinations showed enhanced anti-diabetic effects, including better glucose uptake, enzyme inhibition, and antioxidant activity.	[81]
Mogroside V (MO5), Stevioside (ST), Sucralose (TGS), Erythritol (ERT)	T_2_DM mice	Received MO5, ST, TGS, or ERT (each 48 mg/kg) for 4 weeks	Improved glucose and lipid metabolism via AMP-activated protein kinase (AMPK) activation and peroxisome proliferator-activated receptor α (PPARα)/carnitine palmitoyltransferase I (CPT-1) up-regulation; Enhanced protein synthesis through mammalian target of rapamycin (mTOR)/70 kDa ribosomal protein S6 kinase (P70S6K) pathway.	[63]
MO5, ST, TGS, ERT	T_2_DM mice	Gavage with MO5, ST, TGS, or ERT (48 mg/kg) for 4 weeks.	Improved gut health and inflammation; Enhanced intestinal barrier function.	[64]
Mogrosides, Stevioside, glycyrrhizinic acid, Crude trilobatin, Crude rubusoside	α-glucosidase and α-amylase assays	α-glucosidase and α-amylase inhibition assays	Inhibited both α-glucosidase and α-amylase activities.	[82]

SG, stevia glycoside; MO5, mogroside V; SH, sodium hyaluronate; DPP-4: dipeptidyl peptidase-4; ST, stevioside; TGS, sucralose; ERT, erythritol; T_2_DM, type 2 diabetes mellitus; AMPK, AMP-activated protein kinase; PPARα, Peroxisome proliferator-activated receptor α; CPT-1, Carnitine palmitoyltransferase I; mTOR, Mammalian target of rapamycin; P70S6K, 70 kDa ribosomal protein S6 kinase.

**Table 5:** Application of Mogrosides in the Food.

Main Compounds	Food System	Model	Main Results	References
Monk fruit extract (MFE)	Synbiotic yogurt sweetened with MFE (30 mg/mL) plus inulin (10 mg/mL), fermented with ABY-8 starter culture	type 2 diabetes mellitus (T_2_DM) rats	MFE-synbiotic yogurt improved hepatic lipid-related biomarkers and shifted hepatic metabolic pathways in the T_2_DM context.	[92]
MFE	Synbiotic yogurt wherein MFE served as the sweetener, inulin as the prebiotic source, and ABY-8 as the probiotic starter culture	T_2_DM rats	Improved serum lipid profile; Modulated hepatic AMP-activated protein kinase (AMPK) signaling.	[86]
Monk fruit sweetener and Stevia	Yogurt formulations sweetened with natural sweeteners, replacing sucrose	Sensory study	Showed distinct temporal sensory profiles and acceptable liking.	[93]
Monk fruit sweetener, Stevia, and Sucrose blends	Chocolate milk	Sensory study	Reduced sugar while maintaining acceptable sweetness and sensory quality.	[94]
Monk fruit extract	Strawberry-flavored beverage formulation containing monk fruit extract in water	Healthy males	Increased lunch intake, but not total daily intake	[89]
Monk fruit extract	Strawberry-flavored beverage formulation containing monk fruit extract in water	Healthy males, One day intake	No significant differences in 24 h glucose, total area under the curve (AUC), glucose incremental AUC (iAUC), or glycemic variability compared to sucrose over one day intake.	[90]
Mogroside V, stevia RebM	Lemon-flavored beverage containing a mogroside V/stevia RebM blend	Adults with overweight/obesity	Reduced 2-h insulin iAUC compared to sucrose	[91]

MFE, monk fruit extract; T_2_DM, type 2 diabetes mellitus; AMPK, AMP-activated protein kinase; AUC, area under the curve; iAUC, incremental area under the curve.

## Data Availability

No new data were created or analyzed in this study. Data sharing is not applicable to this article.
